# The French Aging Males' Symptoms (AMS) scale: Methodological review

**DOI:** 10.1186/1477-7525-3-20

**Published:** 2005-03-24

**Authors:** Eric Myon, Nicolas Martin, Charles Taïeb

**Affiliations:** 1 Pierre Fabre SA, 45 place Abel Gance, 92654 Boulogne-Billancourt Cedex, France

**Keywords:** AMS, Aging Males Symptoms, Quality of Life, Questionnaires, Reliability, Validity

## Abstract

**Background:**

The AMS is an internationally used health-related quality of life (HRQoL) scale. The aim of this paper is to provide evidence that the French AMS scale measures HRQoL are as valid as other language versions. We also intend to show whether the application of AMS is really limited to aging males only or not. More generally, we like to demonstrate that the AMS scale is a relevant, validated, sensitive instrument to measure HRQoL and change of symptoms in France.

**Methods:**

We performed a representative survey in France to get data AMS scale data. The French data were compared with existing data from other European countries. Only community-based data were used for this comparison.

**Results and Discussion:**

Reliability (here consistency, Cronbach' s alpha) was found to be good and almost identical with other countries. Validity: the internal structure of the AMS (factorial analysis) was sufficiently comparable with the comparison group of other countries in Europe to conclude that the scale really measures the same phenomenon. The sub-scores and total score correlations (Pearson) were high (r = 0.8–0.9) but only somewhat lower among the sub-scales (r = 0.5–0.7). This suggests that the domains are correlated.

The comparison of the French AMS with the generic quality-of-life scale SF-12 showed a good correlation (Pearson r = 0.48 – 0.51) as reported from other countries. We observed also a good correlation between the AMS scale and the depression scale HAD (Pearson r = 0.62).

The analysis of the AMS structure across age groups showed sufficient similarity to suggest that the AMS is also useful for younger age groups.

**Conclusion:**

The French AMS scale is a standardized HRQoL scale with good psychometric characteristics (reliability, validity) as shown for other international versions. We suggest that the AMS scale could be also used in age groups under 40 years to measure and compare HRQoL in males. Since the application of the AMS in younger age was not investigated before, confirmation in future studies is needed.

## Background

The Aging Males' Symptoms (AMS) scale was originally developed as symptoms profile scale in Germany to evaluate health-related quality of life (HRQoL) [1]. It is a self-administered scale to assess symptoms of aging, to compare the severity of complaints, and to measure therapeutic interventions [1-3].

The AMS scale is an internationally used scale. Currently, translations into 17 languages are available following methodological recommendations for linguistic and cultural adaptation. These versions are available in a published form [2,4,5] and can be downloaded from the Internet .

Norm values of the standardized AMS scores (total score and three domain scores) however were only published for Germany until now. In addition, little is published yet about the comparability of the scale across countries, except one recent paper that provided promising impressions, based however on small numbers in most of the countries [6]. It is important to analyze if the scale measures similarly in different countries or whether one could pool results of clinical studies across countries.

The aim of this paper is to provide evidence that the French AMS scale measures similarly (similar reliability and validity) compared with other language versions. We also intend to show whether the application of AMS is really limited to aging males only or not. More generally, we would like to demonstrate that the AMS scale is a relevant, validated, sensitive instrument to measure HRQoL and change of symptoms in France.

## Methods

The French data are based on representative national sample of French males aged 15 and more years: 963 males underwent a computer-assisted telephone interview that included the French AMS scale [7]. This approach is in full agreement with national ethical regulations and data privacy rules. AMS scores were available for 903 men. The sample was constructed using quotas method by stratification on sex, age, householder's profession, geographic region and population density.

We compared the French data with existing data from other European AMS data built the comparator for this methods paper. Results of this database were published recently [6] as methodological paper of the AMS scale and kindly provided by L.A.J. Heinemann for looking at possible similarities or differences of the French AMS scale. Only community-based European data were used for this comparison: men aged 40 years and more. The bulk of the data came from Germany (96%), UK (2%), and only very small samples (n < 40) from Spain, Portugal, Italy, and Sweden [6]. These samples were combined to build the dataset called "Europe, others" in the following text and tables. The important difference with the French database is the absence of males under 40 years in the "Europe, others" data.

Using these two databases, we were able to review the most fundamental psychometric characteristics of the French AMS, i.e., particularly reliability and some aspects of validity.

It is the first quality requirement for a new scale – here the French AMS – to provide evidence that reliability or replicability is sufficiently good and not different from the same scale in other language regions. We used only the Cronbach's alpha measure. In contrast to systematic and random variation, reliability gives an estimate of method-related measurement error that should be low – what translates in a high consistency coefficient "alpha".

A second basic requirement is validity: whereas reliability can be determined easily with a relatively simple indicator, the validity is almost always a continuous process (construct validation). As a first step, we will analyse the internal structure of the French AMS and analyze the total scale and sub-scales by means of factorial analysis. If the scale is similar to the scales used in other countries in Europe (particularly in Germany), the same domains should be identified and similar factor loadings should be found.

The analyses were done with SPSS Windows 10.0 and STATA 6.0.

## Results and Discussion

### Reliability

Table 1 shows the internal consistency measured with Cronbach's Alpha. The consistency coefficients "alpha" fell between 0.7 and 0.9 in France. The values are not materially different from the alpha values in the combined European sample, i.e. for the AMS total score as well the three subscales. This is indicative for a very acceptable consistency of the French AMS scale – and – compatible with Europe outside France.

**Table 1:** Internal consistency coefficients (alpha) for the AMS scale in France compared with rest of Europe: total score and scores for the psychological, somatic, and sexual sub-scale. Mean and SD of the scale and sub-scales. Community samples.

	**France **(N= 903)		Europe* (N = 5907)	
	Mean (SD)	alpha	Mean (SD)	alpha
Total score	27.7 (9.3)	0.89	28.0 (9.4)	0.90
				
Psychological score	7.6 (2.9)	0.75	7.4 (3.1)	0.82
Somatic score	12.5 (4.7)	0.81	12.6 (4.7)	0.81
Sexual score	7.6 (3.1)	0.76	7.9 (3.4)	0.80

* Database is identical with the methodological review of international data published earlier [6] and kindly provided by L.A. J. Heinemann

In addition, the very close mean values (SD) of the scale's total score and domain scores in the two comparison groups contribute to evidence that the scale does not work differently in France compared to the rest of Europe.

Test-retest correlation coefficients (Pearson's correlation) in a very small sample in France were earlier published [6] and support the above conclusion: Cronbach's alpha for total scale and domains ranged from 0.7 to 0.9 (details in [6]).

### Validity

The first step of validation is usually to multivariately demonstrate the internal structure ("dimensions") of a given scale through factor analysis.

Table 2 shows the comparison of the factorial structure of the French AMS scale and "Europe, others". The structure of the French AMS is not materially different from "Europe, others", i.e. the same three domains were found in the French scale as were described initially for the German original scale. In addition, the proportion of explained variance by the AMS scale is also very similar: 52% and 56% in the French and European analysis (52% in the original German scale in 1999). The loadings of the 17 items on the 3 factors/domains of the French AMS are astonishingly similar with those from rest of "Europe, others".

**Table 2:** Internal structure and domains of the AMS scale. Comparison of community samples of France and other countries of Europe [6]

	N	**FRANCE **903			**EUROPE, other **5907		
	Item Nr.	Psych	Somat	Sex	Psych	Somat	Sex
Burned out	13	0.3	*0.6*		0.7		
Depressive, more	11	0.5	*0.6*		0.8		
Irritability, increased	6	0.7			0.6		
Anxious, more	8	0.6			0.7		
Nervousness, more	7	0.7			0.6		
Joint complaints, more	2		0.6			0.7	
Sweating, increased	3	*0.5*	0.4			0.5	
Sleep, need for more	5		0.7			0.6	
Well-being, impaired	1		0.7			0.7	
Sleep disturbances, more	4		0.6			0.6	
Muscular weakness	10		0.6			0.6	
Physical exhaustion	9		0.6			0.6	
Sexual potency, impaired	15			0.8			0.9
Morning erections, less	16			0.8			0.9
Libido, disturbed	17			0.8			0.8
Passed peak	12			0.5	*0.5*		0.4
Decrease of beard growth	14			0.2	*0.4*		0.3

Factor loadings only depicted if 0.5 or more^# ^in the sub-scales: psychological (psych), somatic (somat), and sexual sub-scale (sex). ^# ^Except if conflicting loadings /not in original domain (italic)

Thus, the French scale does obviously not differ from other versions, and particular the German original, i.e. from this perspective.

However, as reported in a previous publication [6], there are two items that are not particularly helpful in forming the sexual factor: The statement that the beard growth decreased and the feeling to have "passed the peak of life". The loading were (very) low, or showed up in two different domains. Thus, also the French AMS supports the conclusion of an earlier publication [6], " that item 12 ....14 could be eliminated if a new standardization is planned". This however seems not to be feasible in the near future.

Three other items from the psychological domain (burned out, depressive, sweating) have low loadings on more than one domain (somatic domain) in the French AMS or even slightly higher loadings in the "wrong domain". Nevertheless, similarity with the original German AMS scale [6] and "Europe, others" is dominating some differences (including those mentioned above). At least, this seems not to undermine the quality of the French AMS scale. Moreover, it is not likely that these weaknesses influence the use of the scale in practice since individual items are not used – only average scores of the total scale or the three domains. Moreover, it is unlikely to have negative impact on multinational studies, because intra-individual comparisons over time (before/after treatment) are the main criterion that might not be affected very much.

### Sub-scores and total score correlations

The relations among the sub-scales and the aggregate total scale are patterns that are important in the methodological assessment of a scale. In theory, the correlations between subscales (supposed to be independent) should be much lower than the correlations with the total score to which all sub-scales should significantly contribute – the domains are really independent from each other (as suggested by the factorial analysis with orthogonal factors). However, table 4 shows only a somewhat lower Pearson's correlation among sub-scales (r = 0.5–0.7) as compared with correlation of sub-scales with the total score (r = 0.8–0.9).

**Table 4:** Domain score – total score correlations of the AMS scale. Comparison between French and other European community samples.

	**DOMAINS**		
	**Psychological score**	**Somatic score**	**Sexual score**
**France (n = 903)**			
Total score	0.8	0.9	0.8
Psychological score	--	0.7	0.5
Somatic score	--	--	0.6
**Other Europe (n = 5907)**			
Total score	0.8	0.9	0.8
Psychological score	--	0.7	0.5
Somatic score	--	--	0.5

This suggests that the sub-scales are not as independent from each other as one would expect them to be according to the applied model. However, this is again identical for the French AMS and the results obtained in "Europe, others". This however means, that the results of subscales should no be seen in isolation but in the context of other scales, or the result of the total score.

### French AMS and other scales

A sufficient correlation with an internationally well-accepted QoL scale, like SF 12 [7], should be demonstrated also for the French AMS – to check the claim that the AMS scale is a health-related QoL scale.

We applied both scales to all participants of the second half of the French survey (n = 461).

High Pearson's correlation coefficients were observed between the total AMS score and the subscale PCS-12 (r = 0.48; p < 0.0001) as well as with MSC-12 or the SF-12 (r = 0.51; p < 0.0001). For comparison, other authors [3] reported as concurrent validity high correlations of the German AMS scale with the SF-36 for an older population sample (40–70 years): Similar to our results with SF-12, the AMS total score was statistically significant correlated with SF-36: r = 0.49 (p < 0.0001).

Similarly, we observed a good Pearson's correlation with the HAD depression scale [8] and the AMS total score (r = 0.62; p < 0.0001).

We conclude that the French AMS is as able to measure HRQoL as observed for the German version. In addition the AMS result can reflect the degree of depression as measured with the HAD scale.

### AMS applicable for all age groups

The utility of the AMS scale for younger age groups is another question that was often raised but not answered yet due to lack of data. The representative French study included also younger age groups (from 15 years upward). Table 3 (see Additional file) shows the dimensions/domains of the French and the "Europe, others" results in two broad age groups (under/equal 50 vs. over 50 years). The factor loadings of the three domains differ not systematically between the two age groups. It should be kept in mind that the French sample included males from 15 years upward whereas the sample "Europe, others" started with 40 years.

**Table 3:** Internal structure and domains of the AMS scale. Comparison of community samples of France and other countries of Europe [6] in 2 age groups.

	N	**France **903						**Europe, other **5907					
	Age	Under = 50 (n = 617)*			Over 50 (n = 346)			Under = 50 (n = 459)*			Over 50 (5448)		
	Item Nr.	Psych	Somat	Sex	Psych	Somat	Sex	Psych	Somat	Sex	Psych	Somat	Sex
Burned out	13	0.2	*0.6*		0.4	*0.6*		0.7			0.7		
Depressive, more	11	0.6			0.7			0.5	*0.5*		0.8		
Irritability, increased	6	0.6			0.6			0.6			0.6		
Anxious, more	8	0.6			0.7			0.7			0.7		
Nervousness, more	7	0.7			0.7			0.7			0.7		
Joint complaints, more	2		0.6			0.6			0.7			0.8	
Sweating, increased	3	*0.6*	0.2		*0.4*	0.2			0.6			0.5	
Sleep, need for more	5		0.7			0.7			0.6			0.6	
Well-being, impaired	1		0.7			0.6			0.6			0.7	
Sleep disturbances, more	4		0.6			0.6			0.6			0.5	
Muscular weakness	10		0.6			0.7		*0.5*	0.5			0.6	
Physical exhaustion	9		0.7			0.6			0.6			0.6	
Sexual potency, impaired	15			0.7			0.8			0.9			0.9
Morning erections, less	16			0.7			0.8			0.8			0.9
Libido, disturbed	17			0.7			0.8			0.8			0.8
Passed peak	12		*0.5*	0.3	*0.4*	*0.4*	0.3	*0.6*	*0.4*	0.1	*0.5*		0.4
Decrease of beard growth	14			0.4	*0.4*			*0.7*		0.3	*0.4*		0.3

Factor loadings only depicted if 0.5 or more^# ^in the sub-scales: psychological (psych), somatic (somat), and sexual sub-scale (sex).

^#^Except if conflicting loadings /not in original domain (italic).

*Age range different: France 15–50, Europe 40–50

To answer the question if the AMS measures different phenomena in groups under 40 than in "aging males" over 40, we further stratified the French sample and repeated the factor analysis for 10-year age groups (Table 5 [see Additional file]): There is clear evidence from this analysis that the AMS scale measures a similar phenomenon (HRQoL) in all age groups. Of course, the proportion of persons with impaired HRQoL increases with age, i.e. the mean total and domain scores of the AMS increases with 10-year age groups. The mean values of the AMS total score were in the French study: 24.6, 24.7, 27.3, 30.3, and 32.4 scoring points for the age groups under 30, 30–39, 40–49, 50–59, and 60+ years old, respectively. Similarly, the mean scoring points of the three domains increase with age – particularly so in the psychological and somatic domain (data not shown).

**Table 5:** Internal structure and domains of the AMS scale in 10-year age groups. Comparison across age in the French community sample (n = 903).

Total scale: % variance explained		49.6			46.2			52.5			56.7			52.2		
Domains: % variance explained		13.5	20.2	15.9	17.6	18.3	10.3	12.2	23.1	17.2	10.3	28.9	17.4	17.7	20.3	14.1
**Age group (years)**		**Under 30 (n = 197)**			**30–39 (n = 203)**			**40–49 (n = 169)**			**50–59 (n = 154)**			**60+ (n = 180)**		
	Item Nr.	Psych	Somat	Sex	Psych	Somat	Sex	Psych	Somat	Sex	Psych	Somat	Sex	Psych	Somat	Sex

Burned out	13	0.2	*0.5*		0.1	*0.6*		0.3	*0.5*		0.2	*0.7*		0.5	*0.5*	
Depressive, more	11	0.6			0.8			0.5		*0.5*	0.2	*0.8*		0.6		
Irritability, increased	6	0.5			0.6				*0.7*		0.8			0.5		
Anxious, more	8	0.6			0.7			0.7			0.3	*0.6*		0.8		
Nervousness, more	7	0.4	*0.6*		0.3		*0.5*	0.3	*0.7*		0.6			0.7		
Joint complaints, more	2		0.7			0.6			0.5			0.5			0.6	
Sweating, increased	3		0.5		*0.5*	0.1			0.6			0.2	*0.5*	*0.6*	0.2	
Sleep, need for more	5		0.6			0.6			0.6			0.8			0.6	
Well-being, impaired	1		0.6			0.5			0.6			0.8			0.8	
Sleep disturbances, more	4		0.6		*0.4*	0.4			0.6			0.6			0.5	
Muscular weakness	10		0.6			0.5			0.5			0.6			0.7	
Physical exhaustion	9		0.6			0.6			0.6			0.6			0.6	
Sexual potency, impaired	15			0.8		*0.6*	0.2			0.8			0.7			0.8
Morning erections, less	16			0.7		*0.6*	0.3			0.7			0.8			0.8
Libido, disturbed	17			0.7			0.5			0.7			0.8			0.8
Passed peak	12	*0.7*		0.2	*0.5*		0.4		*0.5*	0.4		*0.3*	0.3	*0.4*		0.4
Decrease of beard growth	14			0.7			0.8	*0.7*			*0.7*		0.2	*0.5*		0.4

Factor loadings only depicted if 0.5 or more^# ^in the sub-scales: psychological (psych), somatic (somat), and sexual sub-scale (sex).

^# ^Except if conflicting loadings /not loading in the original domain *(italic*)

Table 5 (see Additional file) shows a tendency that the scale explains an increasing percentage of the total variance with increasing age, although the numbers are in the same ballpark (ranging from 46% to 56%). The factor loading in the somatic and sexual domain seems to be quite stable across age groups. The psychological domain seems to vary more but not clearly associated with increasing age. As discussed above, some items of the scale are more unstable in their association to only one factor and have low loadings in the French study: item 12 and 14 of the sexual domain ("passed peak", beard growth"), item 11 and 13 of the psychological domain ("depressive, "burned out"), and item 3 of somatic domain ("sweating"). In general, the internal structure of the scale is remarkably similar across age groups.

From this result we suggest that the name of the Aging Males' Symptoms scale might be somewhat misleading: The AMS could be applicable in all age groups to measure HRQoL and not only in "aging males", if the results of the French study can be confirmed in other studies.

Thus, we could also calculate reference values across age with the French survey. This will be published in a joint review paper together with reference values from other countries than Germany that became available in recent time. This paper is under preparation.

## Conclusion

The French AMS scale is a standardized HRQoL scale with good psychometric characteristics (reliability, validity) as shown for other international versions. We suggest that the AMS scale should be also used in age groups under 40 to measure and compare HRQoL in males. Since the application of the AMS in younger age was not investigated before, confirmation in future studies is needed.

## Competing interests

We like to declare that the authors EM, NM, CT are working in a pharmaceutical company. But we don't see a conflict of interest in this methodological study.

## Authors' contributions

EM: involved in the co-ordination of the project, contributed to writing the paper; NM: involved in the data analysis and contributed to writing the paper, CT: responsible for the project, contributed to writing the paper;
